# Selective colorimetric sensing of Fe^3+^ by hesperetin-conjugated silver nanoparticles: further investigation of interaction properties with bovine serum albumin

**DOI:** 10.1039/d5ra09319h

**Published:** 2026-04-07

**Authors:** Sushma, Deepti Chauhan, Kalyan Sundar Ghosh

**Affiliations:** a Department of Chemistry, National Institute of Technology Hamirpur Himachal Pradesh 177005 India kalyan@nith.ac.in +91-1972-223834 +91-1972-254104

## Abstract

Hesperetin-conjugated silver nanoparticles (HSP-AgNPs) were prepared using hesperetin as a reducing and stabilizing agent. UV-Vis and FTIR spectroscopy, dynamic light scattering, XPS, XRD and HRTEM were used to confirm the formation of functionalized nanoparticles. HSP-AgNPs displayed notable sensitivity and selectivity in the detection of Fe^3+^ in water. The probe also exhibited strong anti-interference performance against other metal ions. A detection limit (LOD) of 0.41 µM suggested prospective application of HSP-AgNPs in environmental sensing of Fe^3+^. Further, to explore the potential of HSP-AgNPs in biosensing of Fe^3+^, the biodistribution of nanoparticles was checked by studying their interaction with bovine serum albumin (BSA), a model carrier protein. Interactions between BSA and HSP-AgNPs were explored using UV-Vis, steady-state, time-resolved and synchronous fluorescence spectroscopy. HSP-AgNPs caused static quenching of tryptophan fluorescence of BSA. The thermodynamic parameters of binding (Δ*H* and Δ*S*) suggested the predominant involvement of hydrophobic interactions between BSA and nanoparticles.

## Abstract 2

Hesperetin-conjugated silver nanoparticles (HSP-AgNPs) as optical sensor of Fe(iii).

## Graphical abstract

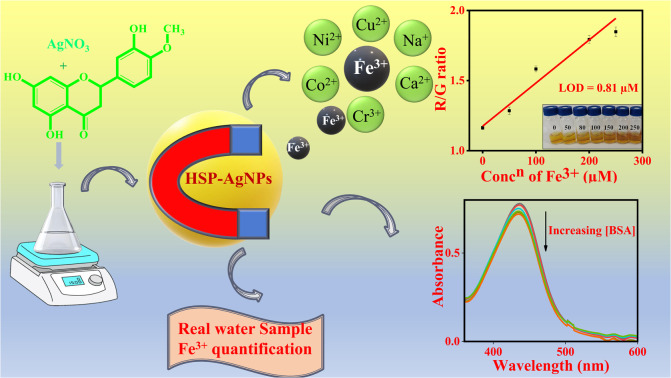

## 1 Introduction

**Fig. 1:**
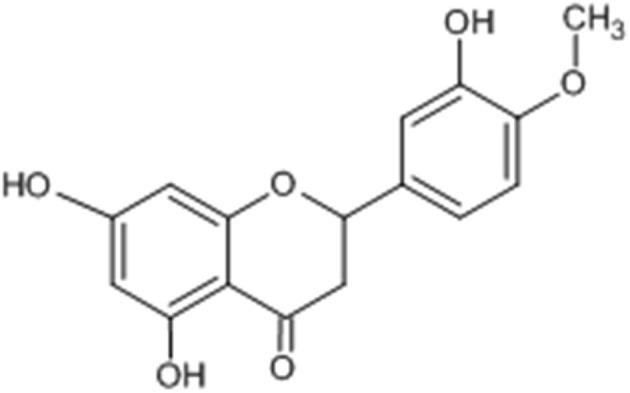
Structure of hesperetin.

Environmental applications of different nanomaterials have extensively been explored in recent times.^1–4^ Among them, silver nanoparticles (AgNPs) attracted additional interest due to their antibacterial and antiviral activities.^5^ The size, surface characteristics, surface functionalizations, morphology and the shape of nanomaterials potentially influence their physicochemical and biological activities. Nowadays, phytochemicals like flavonoids, terpenoids *etc.* are being used in the green synthesis of nanoparticles. Naturally occurring flavonoids have been recognized for their protective roles against oxidation of biomolecules,^6,7^ modulation of enzymatic activity^8^*etc.* One such flavonoid, hesperetin (Fig. 1), commonly found in citrus fruits, played a dual role of reducing agent as well as capping agent during our green synthesis of AgNPs.

Iron is crucially involved in vital physiological processes like oxygen transport and storage. But, imbalance in its concentration can cause anemia, damage of liver and kidney, cardiovascular diseases *etc*.^9^ Therefore, selective and sensitive analytical tools are highly required for quantitative detection of Fe^3+^ in complex matrices like environmental samples, pharmaceutical products, industrial materials *etc.* Fe^3+^ is commonly detected by using atomic absorption spectroscopy, ICP-MS, electroanalytical methods *etc.* But many of these techniques are time-consuming and require expensive equipments along with labor-intensive experimental settings. Hence, development of quick, easy, affordable and reliable techniques for detection and bio-imaging of metal ions will be highly useful.^10–13^ In this context it is pertinent to mention that suitably functionalized AgNPs were reported as colorimetric sensors of different heavy metal ions.^14–18^ AgNPs also demonstrated colorimetric detection capability for Fe^3+^.^19–21^

But in most of the earlier studies, AgNPs-based probes of Fe^3+^ were synthesized using plant extracts which contain several phytochemical molecules. So it is difficult to ascertain that the functionalization of AgNPs by which molecules was actually responsible for exhibiting Fe^3+^ sensing ability. In this contrary, the present work described functionalization of AgNPs with a specific flavonoid hesperetin. To best of our knowledge, specific flavonoid-functionalized AgNPs as sensor of Fe^3+^ may be reported very rarely. Therefore, in this work, hesperetin-conjugated AgNPs (HSP-AgNPs) were developed as a sensor of Fe^3+^ in water. The probe provided a number of benefits, such as simple and fast visual detection, high sensitivity, affordability and convenience in use. Further, to use as efficient biosensor of Fe^3+^, biodistribution of nanoparticles needs to be checked. This was performed by studying interactions between HSP-AgNPs and bovine serum albumin (BSA), a model carrier protein. BSA essentially transports numerous endogenous and exogenous molecules and shares structural and functional similarities with human serum albumin.

## 2 Materials and methods

### 2.1 Materials

NaCl, KCl, CaCl_2_, CrCl_3_, Al_2_(SO_4_)_3_, MnCl_2_, FeCl_3_, HgCl_2_, Co(CH_3_COO)_2_, Ni(NO_3_)_2_, CuCl_2_, ZnCl_2_, Pb(CH_3_COO)_2_, Cd(NO_3_)_2_ were used to prepare the solutions of metal ions. Hesperetin was from Merck and rest other chemicals were from SRL India and Himedia. Type I ultrapure water was used in all the experiments. BSA solution was prepared in 50 mM phosphate buffer (pH 7.4). Fourier transform infrared (FTIR) spectrum was recorded in IRAffinity-1S (Shimadzu). HRTEM image was recorded by using transmission electron microscope (Philips, CM100). Hydrodynamic diameter and zeta potential of the nanoparticles were determined using dynamic light scattering (Malvern Zetasizer). XPS and XRD measurements were carried out respectively using PHI Quantes Scanning Dual X-ray Photoelectron Microprobe and Cu Kα radiation (*λ* = 0.15406 nm) over a 2*θ* range of 5–80°. UV-Vis and fluorescence spectra were recorded using Motras and Shimadzu RF-5300 spectrophotometer respectively.

### 2.2 Synthesis of hesperetin-coated silver nanoparticles

Following an earlier reported method,^22^ hesperetin was dissolved in 0.01 mM NaOH at 80 °C. 10 ml of 1 mM silver nitrate solution was mixed with 10 ml hesperetin solution (1 mM) and the mixture was heated at 70 °C for 20 minutes. Appearance of yellow color indicated reduction of Ag^+^ to elemental silver (Ag^0^). The pH of the synthesized HSP-AgNPs was found ∼7 and the nanoparticles were further stored at 4 °C.

### 2.3 Colorimetry and UV-visible studies for sensing of metal ions by HSP-AgNPs

Different cations (Na^+^, K^+^, Ca^2+^, Cr^3+^, Al^3+^, Mn^2+^, Fe^3+^, Hg^2+^, Co^2+^, Ni^2+^, Cu^2+^, Zn^2+^, Pb^2+^, Cd^2+^) were added individually to HSP-AgNPs and the color was noted. Also, the absorption spectra of HSP-AgNPs were recorded before and after addition of each cation (350 µM) to the nanoparticles. Also, the image of probe solution before and after addition of different concentration of Fe^3+^ was captured using a smartphone (Redmi 9 Miui Global 12.5.6, Android 11 model with 13-megapixel rear camera) at 10 cm distance under uniform natural daylight. Further, *R*, *G* and *B* values of the images were measured through *Colorimeter app.* The ratio of *R* and *G* intensity was plotted against Fe^3+^ concentration.

### 2.4 Studies on interactions between HSP-AgNPs and BSA

#### 2.4.1 UV-visible

To a solution of HSP-AgNPs (0.1 nM), BSA was added consecutively up to a concentration of 154 nM. UV-Vis spectra were recorded after each addition of BSA.

#### 2.4.2 Steady-state fluorescence

To a solution of BSA (4 µM), HSP-AgNPs (0.25 to 4.06 pM) was added at four different temperatures (281, 289, 297 and 305 K). Emission spectra of BSA were recorded using an excitation at 295 nm after each addition of HSP-AgNPs. The recorded fluorescence intensity (*F*_recorded_) values were corrected for inner filter effect according to following equation, where *A*_ex_ and *A*_em_ are the absorbance at the excitation and emission wavelengths respectively. Corrected intensities (*F*_corrected_) were used for further calculations.
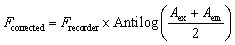


Stern–Volmer plot was used to analyze the quenching data in order to calculate the quenching constant (*K*_sv_). Binding parameters were calculated from double logarithmic plot. Thermodynamic parameters (Δ*H*, Δ*S* and Δ*G*) were determined from van't Hoff's plot.

#### 2.4.3 Synchronous fluorescence

Synchronous fluorescence spectra of BSA (4 µM) were recorded at varying concentrations of HSP-AgNPs (0.25 to 4.06 pM). The excitation and emission monochromator wavelength difference (Δ*λ*) was maintained at 60 nm for tryptophan (Trp) residue and at 15 nm for tyrosine (Tyr) residue.

#### 2.4.4 Fluorescence lifetime analysis

A time-correlated single-photon counting (TCSPC) system (Fluorocube-01-NL, IBH) was used to measure the excited-state lifetime of tryptophan residues in BSA (10 µM) in absence and the presence of HSP-AgNPs (0.9 pM). A 280 nm NanoLED source was used for excitation. In order to remove polarization artifacts, fluorescence emission was measured at the magic angle (54.7°). IBH DAS software (version 6) was used to analyze the data. Detail experimental setup and data analysis has been described elsewhere.^23^ Reduced chi-square (*χ*^2^) values and residual analysis were used to assess the decay curve fitting quality.

## 3 Results and discussion

**Fig. 2:**
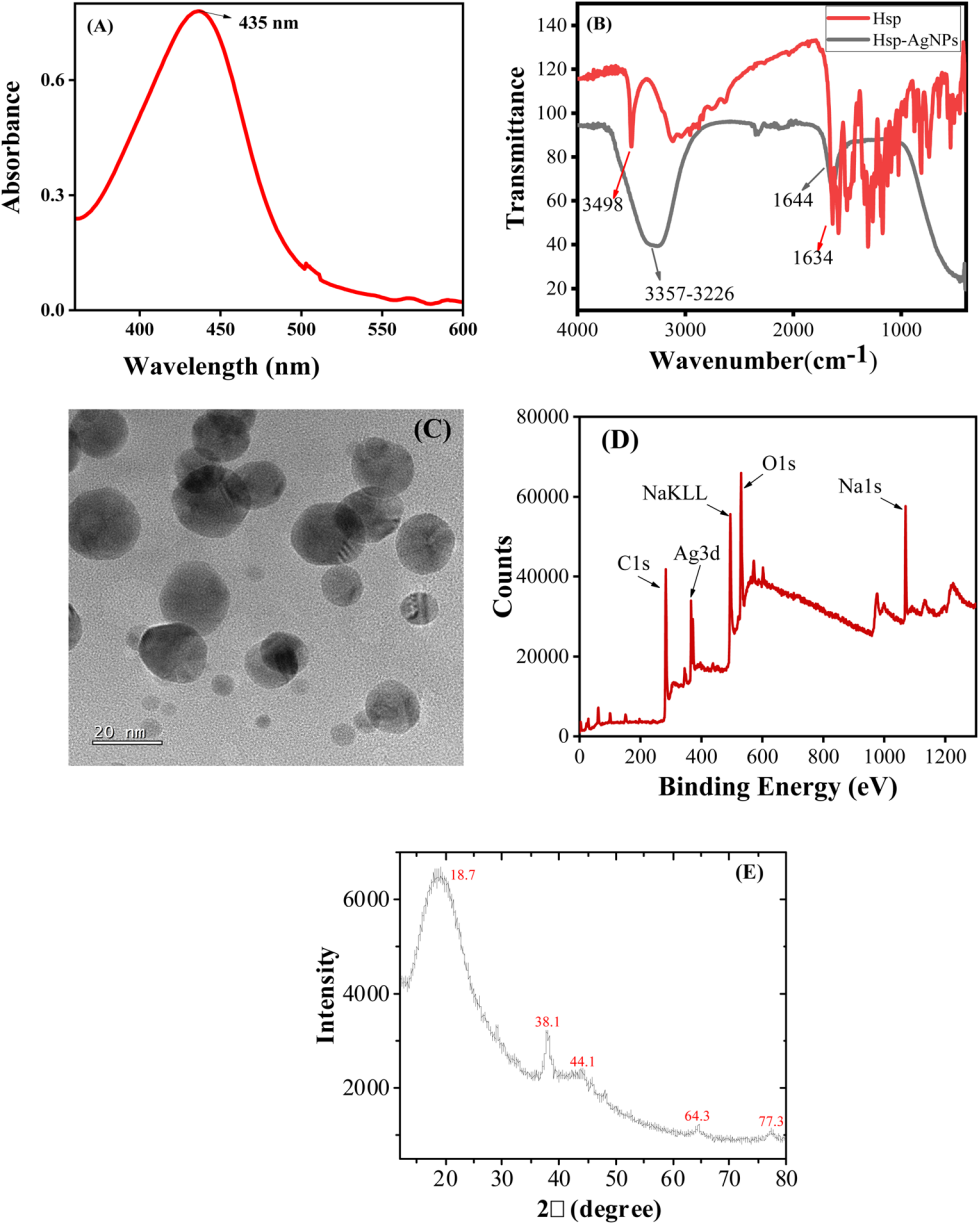
(A) UV-Vis absorbance spectrum of HSP-AgNPs; (B) FTIR spectrum of HSP-AgNPs; (C) TEM image of HSP-AgNPs; (D) XPS full-survey spectrum of HSP-AgNPs; (E) X-ray diffraction pattern of HSP-AgNPs.

UV-Vis spectroscopic analysis of the synthesized HSP-AgNPs revealed a distinct surface plasmon resonance (SPR) absorption peak positioned at 435 nm (Fig. 2A), which supported successful synthesis of HSP-AgNPs. The FTIR spectrum of synthesised HSP-AgNPs was recorded and compared with that of hesperetin (Fig. 2B). The sharp –OH stretching band at 3498 cm^−1^ of hesperetin was shifted at lower wavenumber with significant broadening of the band (3357–3226 cm^−1^) in HSP-AgNPs. The C=O stretching band of hesperetin (1634 cm^−1^) was shifted toward higher wavenumber (1644 cm^−1^) in HSP-AgNPs. These noticeable shifts in FTIR peaks confirmed successful capping of AgNPs by hesperetin. The shape of the synthesized HSP-AgNPs was visualized using TEM. The nanoparticles exhibited predominantly spherical morphology in the TEM micrograph (Fig. 2C). The average particle size of HSP-AgNPs was calculated ∼14 nm from size distribution histogram (SI Fig. S1A). Existence of clear crystal lattice planes in the HRTEM image (SI Fig. S1B) confirmed substantial crystallinity in HSP-AgNPs. The SAED pattern (SI Fig. S1C) demonstrated dotted diffraction rings suggested polycrystalline nature of the nanoparticles. The concentration of nanoparticles was calculated using the average particle size of HSP-AgNPs following the method^24^ described in the Supplementary Materials.

The size of HSP-AgNPs was also determined by using dynamic light scattering (DLS) technique (SI Fig. S2A). The hydrodynamic diameter of the nanoparticles measured by DLS was found ∼25 nm. The zeta potential of the nanoparticles was determined as −22.1 mV (SI Fig. S2B), which indicated good stability of HSP-surface functionalized AgNPs. Due to large negative zeta potential of the nanosuspension, the nanoparticles would repel each other to reduce the possibility of agglomeration. Distinct XPS peaks of HSP-AgNPs at 286, 369 and 534 eV (Fig. 2D) were assigned as C1s, Ag3d and O1s, which suggested successful synthesis and capping of AgNPs with hesperetin. The XRD pattern of HSP-AgNPs (Fig. 2F) showed characteristic diffraction peaks at 2*θ* ≈ 38°, 44°, 64°, and 77°, corresponding to the (111), (200), (220), and (311) planes of face-centered cubic metallic silver. A broad diffraction band centred ∼18.7° was attributed to the amorphous organic phase of hesperetin capping, confirming successful surface functionalization. Lower intensity and broadening of Ag XRD peaks was due to nanoscale crystallite size and possible surface strain induced by flavonoid interactions.

**Fig. 3:**
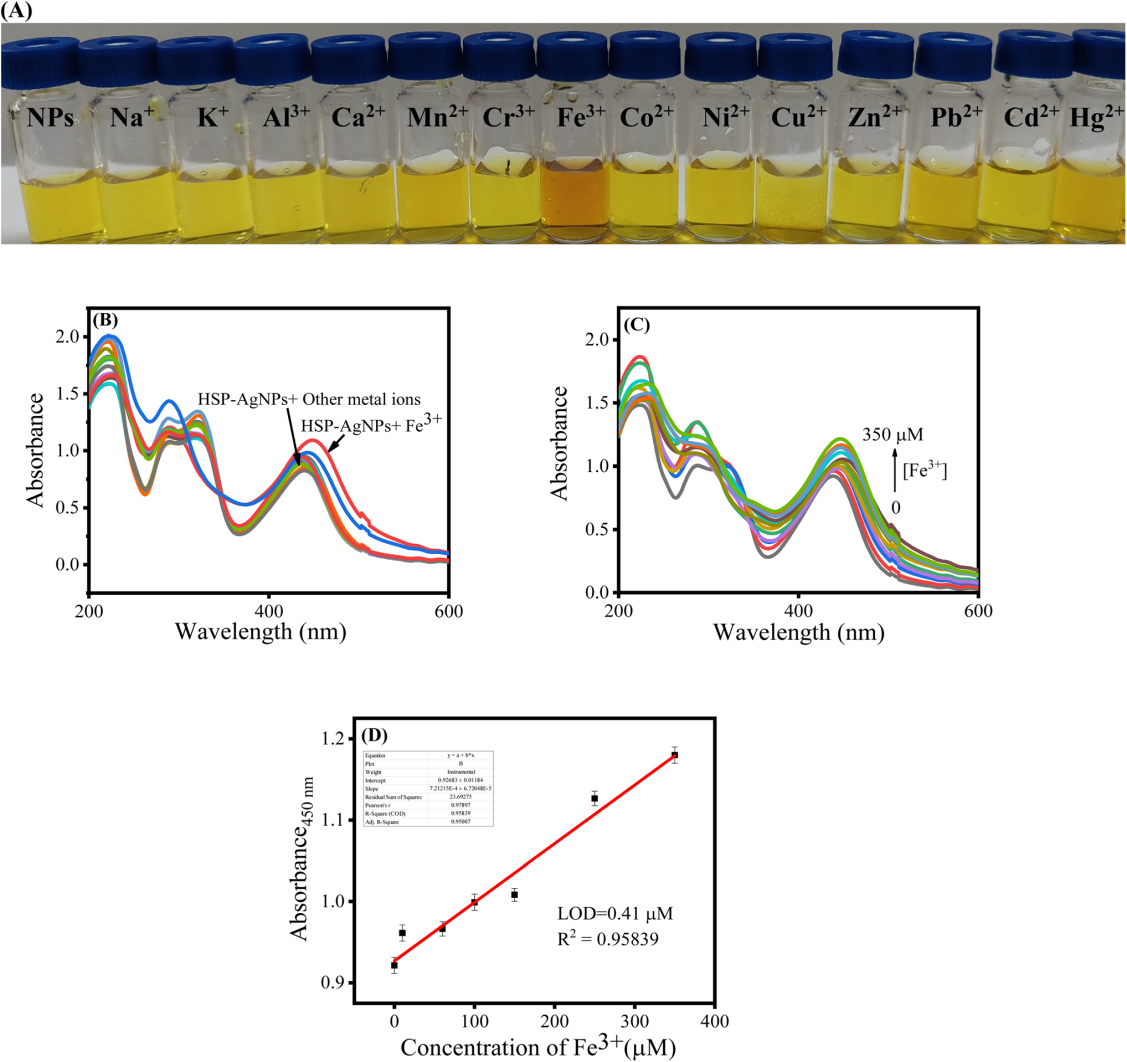
(A) HSP-AgNPs solution observed in natural light before and after the individual addition of various metal ions (350 µM); (B) absorption spectra of HSP-AgNPs in absence and the individual presence of different metal ions (350 µM); (C) UV-vis spectra of HSP-AgNPs in the presence of Fe^3+^ ions (0–350 µM); (D) calibration curves for the colorimetric sensing of Fe^3+^ by HSP-AgNPs (pH: 7, Temp.: 27 °C).

Different metal ions (Na^+^, K^+^, Al^3+^, Ca^2+^, Mn^2+^, Cr^3+^, Fe^3+^, Co^2+^, Ni^2+^, Cu^2+^, Zn^2+^, Pb^2+^, Cd^2+^ and Hg^2+^) in aqueous media at a concentration of 350 µM were added individually to HSP-AgNPs to assess the colorimetric recognition capability of nanoparticles. Addition of Fe^3+^ changed the color of the nanoparticles from yellow to orange (Fig. 3A). Other metal ions did not cause any notable change in color. The selectivity of HSP-AgNPs for colorimetric detection of Fe^3+^ was established. Recognition of Fe^3+^ by HSP-AgNPs was further demonstrated by using UV-Vis spectroscopy. A red-shift from 435 to 450 nm in the absorption spectrum of HSP-AgNPs along with increase in absorbance was observed upon addition of Fe^3+^ to the nanoparticles (Fig. 3B). Coordination of Fe^3+^ with the phenolic –OH groups of hesperetin present on the surface of nanoparticles might cause this spectral shift. Sensing performance of HSP-AgNPs was quantified by adding different concentrations (0 to 350 µM) of Fe^3+^ to the nanoparticles. Continuous increase in absorbance (at 450 nm) with increase in the concentration of Fe^3+^ was noticed (Fig. 3C). The limit of detection (LOD) of Fe^3+^ by HSP-AgNPs was determined as 0.41 µM from Fig. 3D, using the equation LOD = 3*σ*/*s*^25–27^ (where *σ* is the standard deviation in absorbance of the blank solution and *s* is the slope of the calibration plot). A good linear relationship between absorbance and the concentration of Fe^3+^ ions was observed in the range of 0 to 350 µM. Sensitivity of the detection was also determined from colrimetric assay by using a smartphone. The *R*/*G* intensity ratio of HSP-AgNPs increased linearly with increase in the concentration of Fe^3+^ (SI Fig. S3). Concentration-dependent modulation of surface plasmon resonance of the nanoparticles arose due to specific interactions between Fe^3+^ ions and surface-bound functional groups of hesperetin. This ratiometric response also enabled reliable quantitative detection of Fe^3+^ with a low limit of detection (0.81 µM).

HSP-AgNPs were also compared with earlier reported colorimetric probes made of silver nanoparticles in terms of Fe^3+^ sensing capability (Table 1). Wider linear range of detection of Fe^3+^ and quite low LOD value of HSP-AgNPs were noticed than most of the earlier reported AgNPs. According to the guidelines of the World Health Organization, the acceptable concentration of iron in drinking water is 0.3 mg L^−1^ (5.37 µM).^28^ The LOD achieved in the present study (0.41 µM) was significantly lower than the regulatory threshold, indicating that the developed sensing platform was capable for selective and efficient detection of Fe^3+^ even at a concentration well below the permissible limit.

**Table 1:** Comparison of the analytical performance of HSP-AgNPs with earlier reported colrimetric AgNPs-based sensors of Fe^3+^

AgNPs	Linear range (µM)	LOD (µM)	Ref.
Quinone-capped	1–100	1	19
*N*-acetyl-l-cysteine-stabilized	0.08–8	0.08	29
*B. variegate* extract-stabilized	6–100	2.08	30
Synthesized using extract of *S. cumini*	10–100	1.2	31
4-ATP-CT-EDTA-L-TA-conjugated	6.63–1800	2.32	32
Synthesized using sapindus mukorossi pericarp extract	0–100	5	33
Synthesized using leaf extract of *Sonchus arvensis* L.	—	1000	34
Chitosan capped	1–500	0.53	35
β-alanine dithiocarbamate-conjugated	—	6.18	36
Hesperetin-ceonjugated	0–350	0.41	This work

**Fig. 4:**
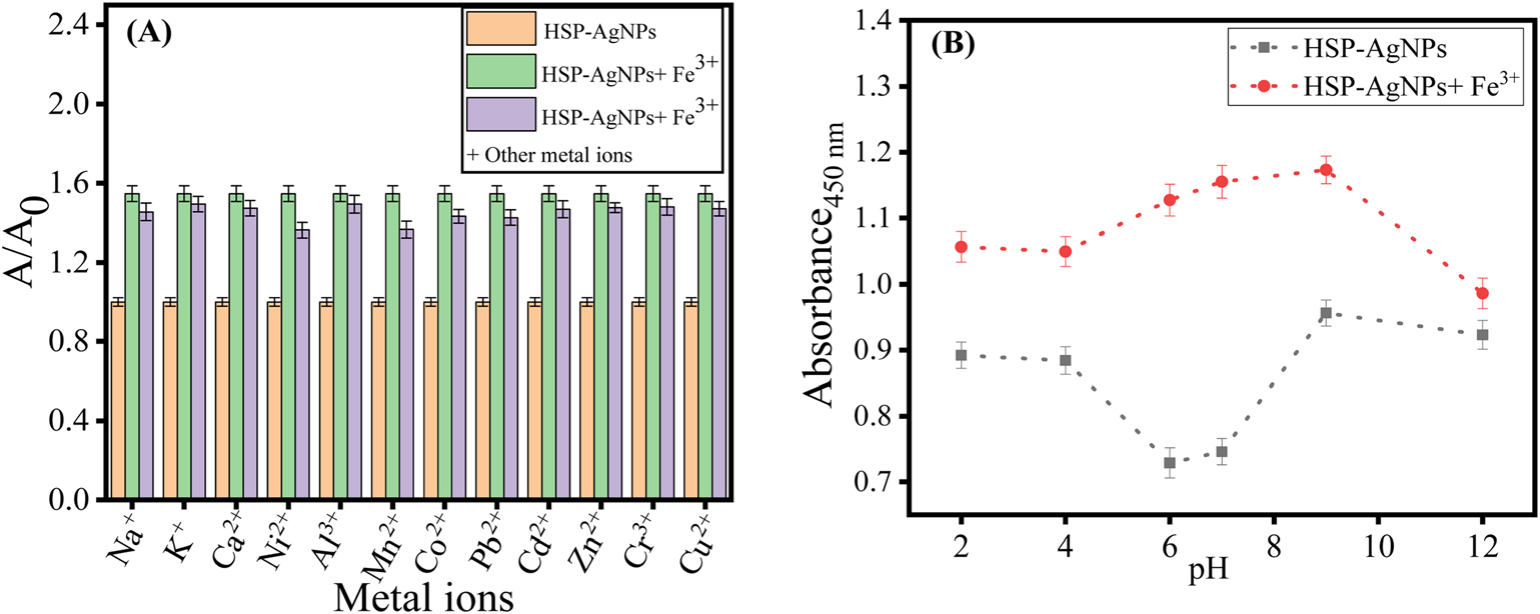
(A) *A*/*A*_0_ ratio of HSP-AgNPs measured at 450 nm in the presence of Fe^3+^ (350 µM) and other metal ions (350 µM); (B) absorbance (at 450 nm) of HSP-AgNPs after addition of Fe^3+^ (350 µM) at different pH (Temp.: 27 °C).

To verify the interfering effect of other metal ions on the detection capability of HSP-AgNPs, 350 µM of Na^+^, K^+^, Ca^2+^, Cr^3+^, Al^3+^, Mn^2+^, Co^2+^, Ni^2+^, Cu^2+^, Zn^2+^, Pb^2+^, Hg^2+^and Cd^2+^ were added individually to Fe^3+^-HSP-AgNPs (Fe^3+^ concentration 350 µM). After addition of these metal ions, the absorbance (at 450 nm) was recorded. Insignificant change in the absorbance of Fe^3+^-HSP-AgNPs after addition of other metal ions suggested strong anti-interference ability of the probe for colorimetric detection of Fe^3+^ (Fig. 4A). The effect of pH on HSP-AgNPs and its sensing ability of Fe^3+^ was also studied (Fig. 4B). A visible variation in color of HSP-AgNPs from yellow to orange was observed with the change in pH (SI Fig. S4). The change in color of nanoparticles at higher pH would be attributed to deprotonation of phenolic –OH groups of hesperetin, which were used in capping of the surface of AgNPs. Therefore, change in pH might alter the surface charge density and local electronic environments, which modulated the surface plasmon resonance and hence the color of AgNPs. In the pH range from 2 to 9, the absorbance of HSP-AgNPs was increased significantly (at 450 nm) on addition of Fe^3+^. In strong alkaline medium (pH > 10), no notable increase in absorbance was observed. Therefore, HSP-AgNPs can act as a reliable colorimetric sensor of Fe^3+^ in the pH range of 2 to 9.

### 3.1 Real sample analysis using HSP-AgNPs

HSP-AgNPs were further used to determine the concentration of Fe^3+^ in different types of water samples. Using ICP-OES technique, Fe^3+^ was not detected in any sample. Then these samples were spiked with a known concentration of Fe^3+^ and further used for quantitative analysis by HSP-AgNPs. After addition of these samples to the nanoparticles, the absorbance was recorded at 450 nm and the concentration of Fe^3+^ was determined from the calibration curve. The concentration determined by HSP-AgNPs and the percentage recovery have been mentioned in Table 2, which was found satisfactory.

**Table 2:** Analysis of real water sample using HSP-AgNPs for quantitative estimation of Fe^3+^

Sample for Fe^3+^ analysis	Spiked Fe^3+^ (µM)	Fe^3+^ concentration determined by HSP-AgNPs (µM)	Recovery % for Fe^3+^
Drinking water	43	43.13 ± 0.21	100.30 ± 0.72
	68	68. 42 ± 0.45	100.6 ± 0.34
	113	113.45 ± 0.60	100.3 ± 0.04
	164	164.50 ± 0.62	100.30 ± 0.05
	193	191.4 ± 0. 91	99.1 ± 0.36
	313	317.8 ± 0.97	101.5 ± 0.51
Tap water	25.0	22.6 ± 0.32	90.4 ± 0.36
	73.0	76.52 ± 0.51	104.8 ± 0.72
	129.0	126.35 ± 0.21	97.94 ± 0.42
	192.0	194.18 ± 0.61	101.13 ± 0.34
	268.0	265.42 ± 0.71	99.03 ± 0.65
	305.0	299.52 ± 0.34	98.20 ± 0.03
Underground water	8.6	9.10 ± 0.23	105.81 ± 0.05
	81.4	80.60 ± 0.56	99.01 ± 0.43
	139.6	141.52 ± 0.86	101.37 ± 0.82
	186.7	184.32 ± 0.28	98.72 ± 0.63
	276.9	279.12 ± 0.86	100.8 ± 0.36
	346.7	348.85 ± 2.32	100.6 ± 0.69

### 3.2 Studies on interactions between HSP-AgNPs and BSA

**Fig. 5:**
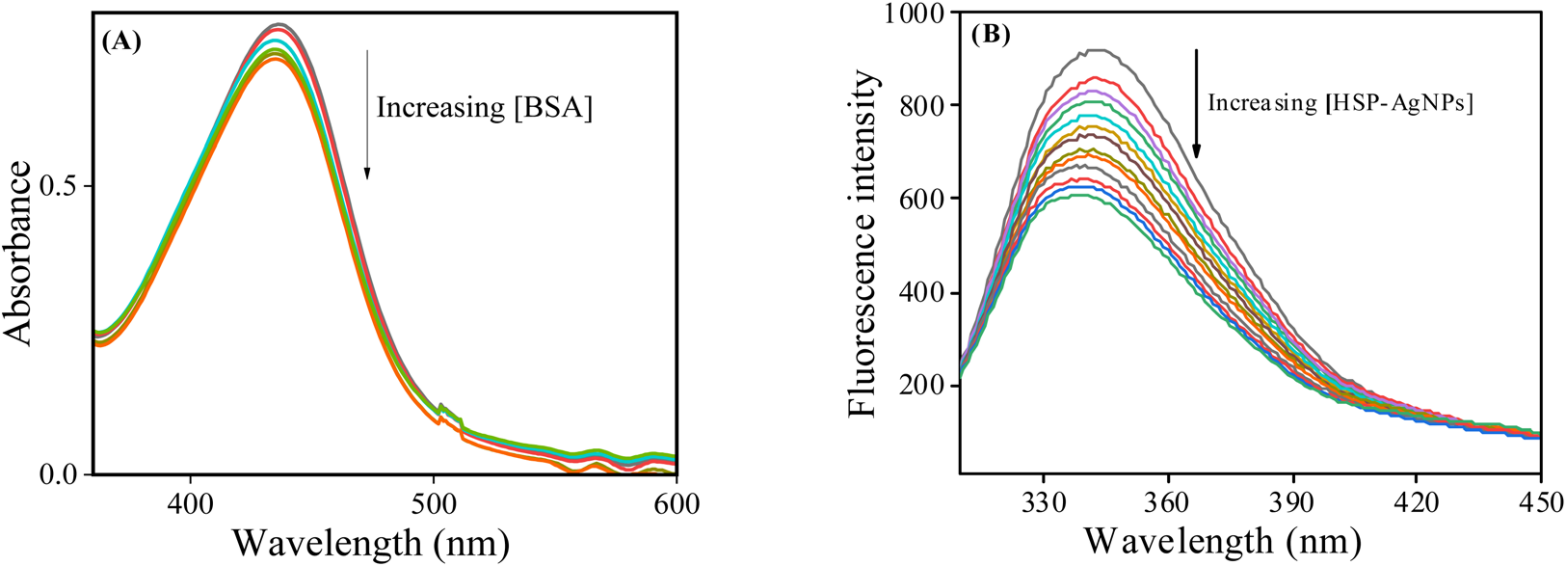
(A) UV-vis spectra of HSP-AgNPs (0.1 nM) in absence and presence of BSA (14.9 to 154 nM), (pH: 7, Temp.: 27 °C); (B) tryptophan fluorescence spectra of BSA (4 µM) in absence and the presence of HSP-AgNPs (0.25 to 4.06 pM) at 289 K, *λ*_ex_: 295 nm, pH: 7.

A hypochromic shift of the SPR band of HSP-AgNPs at 440 nm was observed on addition of BSA (Fig. 5A). As BSA did not have any absorbance at 440 nm, it clearly suggested that the surface of HSP-AgNPs was progressively getting coated by the protein molecules similar to an observation reported earlier.^37^ On the other hand, the tryptophan (Trp) fluorescence intensity of BSA at 340 nm (upon excitation at 295 nm) was gradually quenched after consecutive addition of HSP-AgNPs (Fig. 5B) at four different temperatures. As HSP-AgNPs did not fluoresce at this wavelength, the observed quenching was probably due to the formation of stable complex between HSP-AgNPs and BSA, which was further confirmed from time-resolved fluorescence.

**Fig. 6:**
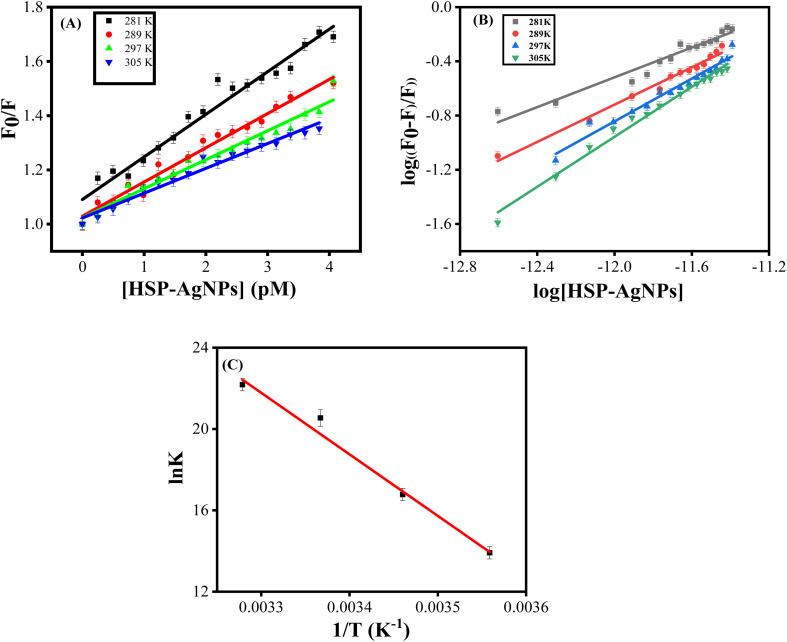
(A) Stern–Volmer plot for quenching of Trp fluorescence of BSA by HSP-AgNPs and (B) double logarithmic plot at 281, 289, 297, 305 K, [BSA] = 4 µM, [HSP-AgNPs] = 0.25 to 4.06 pM, *λ*_ex_ = 295 nm; (C) van't Hoff plot for binding of HSP-AgNPs with BSA. The pH was maintained at 7.

Furthermore, the quenching and binding parameters (Table 3) were calculated using Stern–Volmer and double logarithmic plots (Fig. 6A and B). Linear nature of Stern–Volmer plots indicated either static or dynamic quenching of fluorescence. *K*_SV_ exhibited an inverse correlation with temperature, suggesting that the fluorescence quenching of BSA was due to ground–state complex formation. This temperature dependence is characteristic of static quenching, where non-fluorescent complexes are formed between the fluorophore and quencher in the ground state.

**Table 3:** Quenching and binding parameters between HSP-AgNPs and BSA

Temp (K)	*K* (10^6^ M^−1^)	*n*	*K*_sv_ (10^11^ M^−1^)	Δ*G* (kJ mol^−1^)	Δ*H* (kJ mol^−1^)	Δ*S* (J K^−1^ Mol^−1^)
281	1.10	0.6	1.57	−32.6	254.8	1023.2
289	19.23	0.6	1.20	−40.8		
297	833.68	0.8	0.96	−49.0		
305	4275.62	0.8	0.80	−57.2		

The binding constant (*K*), which reflected the stability of the BSA-HSP-AgNPs complex, was found to be in the range of 10^6^–10^10^ M^−1^. High binding affinity implied strong interactions between BSA and HSP-AgNPs. Notable increase in the binding constant with rising temperature suggested predominant role of hydrophobic interactions in the binding process as also noticed earlier in the interactions of AgNPs and lysozyme.^38^ Additionally, the number of binding sites was increased with temperature, likely due to structural changes in BSA that exposed more binding regions for nanoparticle interaction.

Thermodynamic parameters (Δ*H* and Δ*S*), derived from the van't Hoff plot (Fig. 6C), provided insight into the nature of the binding. The sign and magnitude of Δ*H* and Δ*S* were used to infer the nature of the binding forces.^39^ The spontaneous nature of the binding process was confirmed from the negative values of Δ*G* at all temperatures. Positive value of both Δ*H* and Δ*S* suggested that the interaction was entropy-driven and endothermic. Therefore, binding between BSA and HSP-AgNPs was primarily controlled by hydrophobic interactions.

**Fig. 7:**
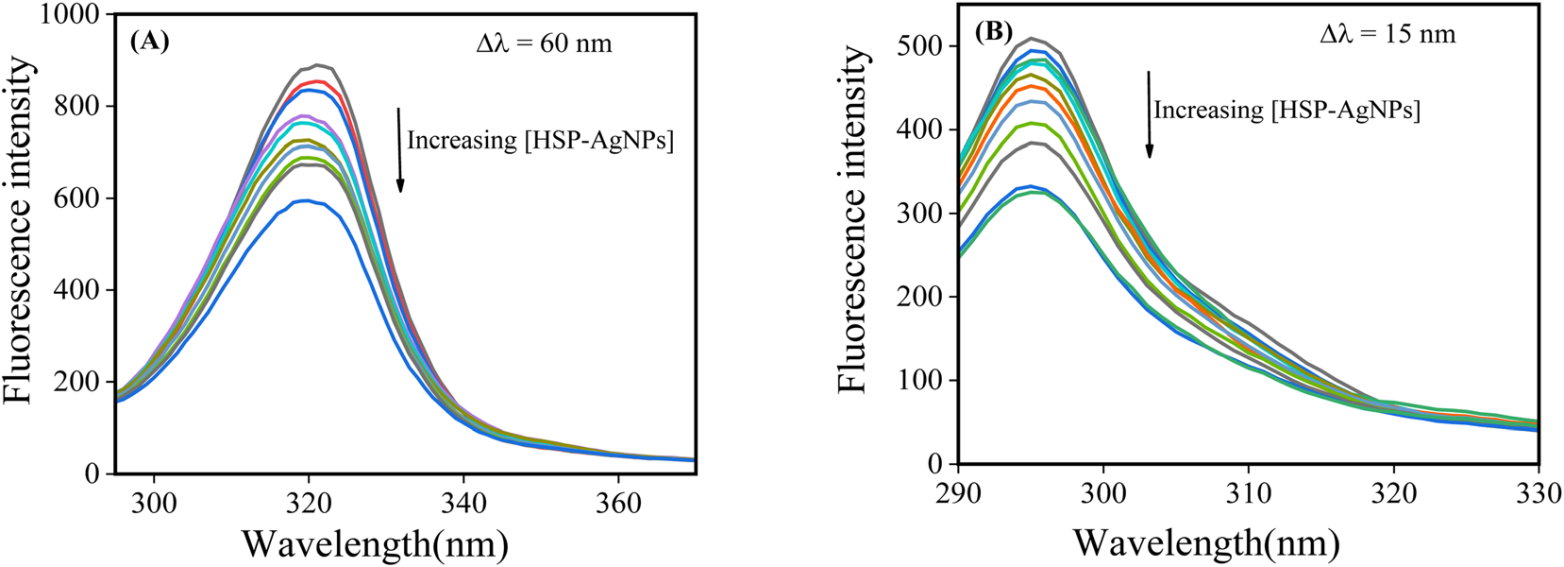
Synchronous fluorescence spectra of BSA at Δλ (A) 60 nm and (B) 15 nm after consecutive addition of HSP-AgNPs (0.25 to 4.06 pM) to BSA (4 µM), (pH: 7, Temp.: 27 °C).

Synchronous fluorescence spectroscopy was used to study any change in the microenvironment of tryptophan (Trp) and tyrosine (Tyr) residues of the protein by adjusting the wavelength difference (Δ*λ*) between the excitation and emission monochromator as 60 and 15 nm respectively. In case of Δ*λ* = 60 nm, slight blue shift was noticed with increase in the concentrations of HSP-AgNPs (Fig. 7A). This reflected decrease in polarity around the Trp residues after addition of nanoparticles. This was consistent with the findings of steady-state fluorescence and implied that HSP-AgNPs interacted close to the Trp residues of BSA. At Δ*λ* = 15 nm, no discernible change was noticed (Fig. 7B).

**Fig. 8:**
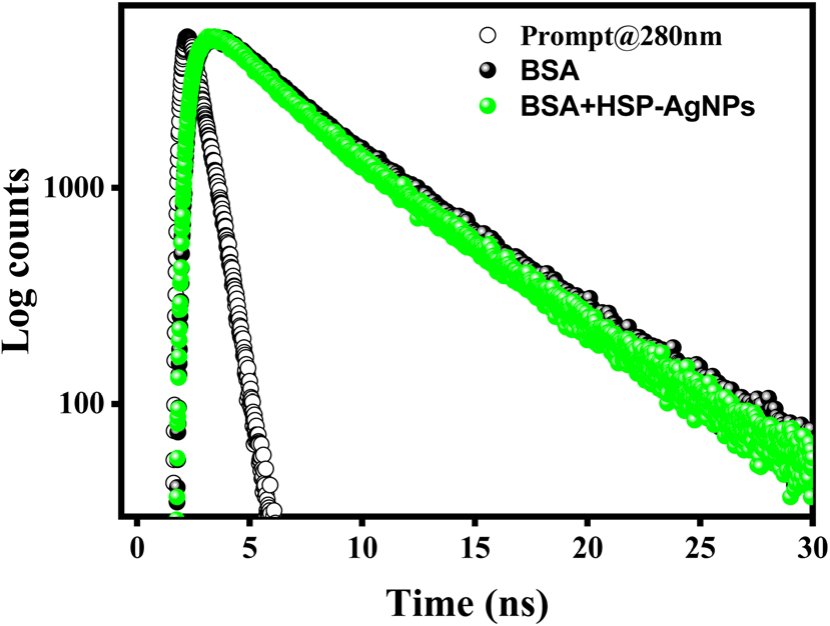
Time-resolved Trp fluorescence decay of BSA (10 µM) in absence and the presence of HSP-AgNPs (0.9 pM), (pH: 7, Temp.: 27 °C).

To further validate the mechanism of Trp fluorescence quenching by HSP-AgNPs, time-resolved fluorescence decay measurements were performed. Trp fluorescence decay of BSA in the presence and absence of HSP-AgNPs were presented in Fig. 8. Fitting data of Trp fluorescence decay were mentioned in Table 4. Excited state life time of Trp of BSA in absence and the presence of HSP-AgNPs were 2.61 and 2.51 ns respectively. Negligible change in life time confirmed static quenching of Trp fluorescence of BSA by HSP-AgNPs.

**Table 4:** Fitting of fluorescence decay of Trp of BSA in absence and the presence of HSP-AgNPs

	*χ* ^2^	*a*_1_	*τ*_1_ (ns)	*a*_2_	*τ*_2_ (ns)	*a*_3_	*τ*_3_ (ns)	*τ*_avg_ (ns)
BSA	1.084	0.35	1.638	0.37	1.025	0.28	5.876	2.605
HSP-AgNPs-BSA	1.026	0.38	1.685	0.37	1.051	0.26	5.838	2.513

## 4 Conclusion

Silver nanoparticles (HSP-AgNPs) were conjugated with hesperetin during their synthesis where hesperetin acted as the reducing as well as stabilizing agent. Spectroscopic and microscopic studies confirmed the formation of conjugated silver nanoparticles. HSP-AgNPs demonstrated remarkable sensitivity and selectivity in the detection of Fe^3+^ in water with LOD value of 0.41 µM. Further, interactions between BSA and HSP-AgNPs were studied using various spectroscopic techniques. HSP-AgNPs caused static quenching of tryptophan fluorescence of BSA with predominant involvement of hydrophobic association in the binding process.

## Author contributions

The work was designed by Sushma and KSG. Experimental data were acquired by Sushma and DC. Analysis and interpretation of the experimental results, drafting and further editing revision of the manuscript were done by Sushma, DC and KSG.

## Conflicts of interest

All the authors declare that there is no conflicts of interest.

## Supplementary Material

RA-016-D5RA09319H-s001

## Data Availability

The data supporting this article have been included in the manuscript and supplementary information (SI). Supplementary information is available. See DOI: https://doi.org/10.1039/d5ra09319h.
