# Supplementary material for: Selective colorimetric sensing of Fe3+ by hesperetin-conjugated silver nanoparticles: further investigation of interaction properties with bovine serum albumin
Source: RSC Adv. 2026 Apr 7;16(19):17497–506. doi: 10.1039/d5ra09319h (PMC13055437; doi:10.1039/d5ra09319h)
Supplement: RA-016-D5RA09319H-s001 [file RA-016-D5RA09319H-s001.pdf]

Supplementary Information (SI) for RSC Advances.
This journal is © The Royal Society of Chemistry 2026

Supplementary Material

Selective Colorimetric Sensing of $Fe^{3+}$by Hesperetin-Conjugated Silver Nanoparticles: Further Investigation of Interaction Properties with Bovine Serum Albumin

Sushma, Deepti Chauhan and Kalyan Sundar Ghosh*

Department of Chemistry, National Institute of Technology Hamirpur, Himachal Pradesh 177005, India

***Corresponding Author:** KSG: Tel: +91-1972-254104; Fax: +91-1972-223834; e-mail: kalyan@nith.ac.in

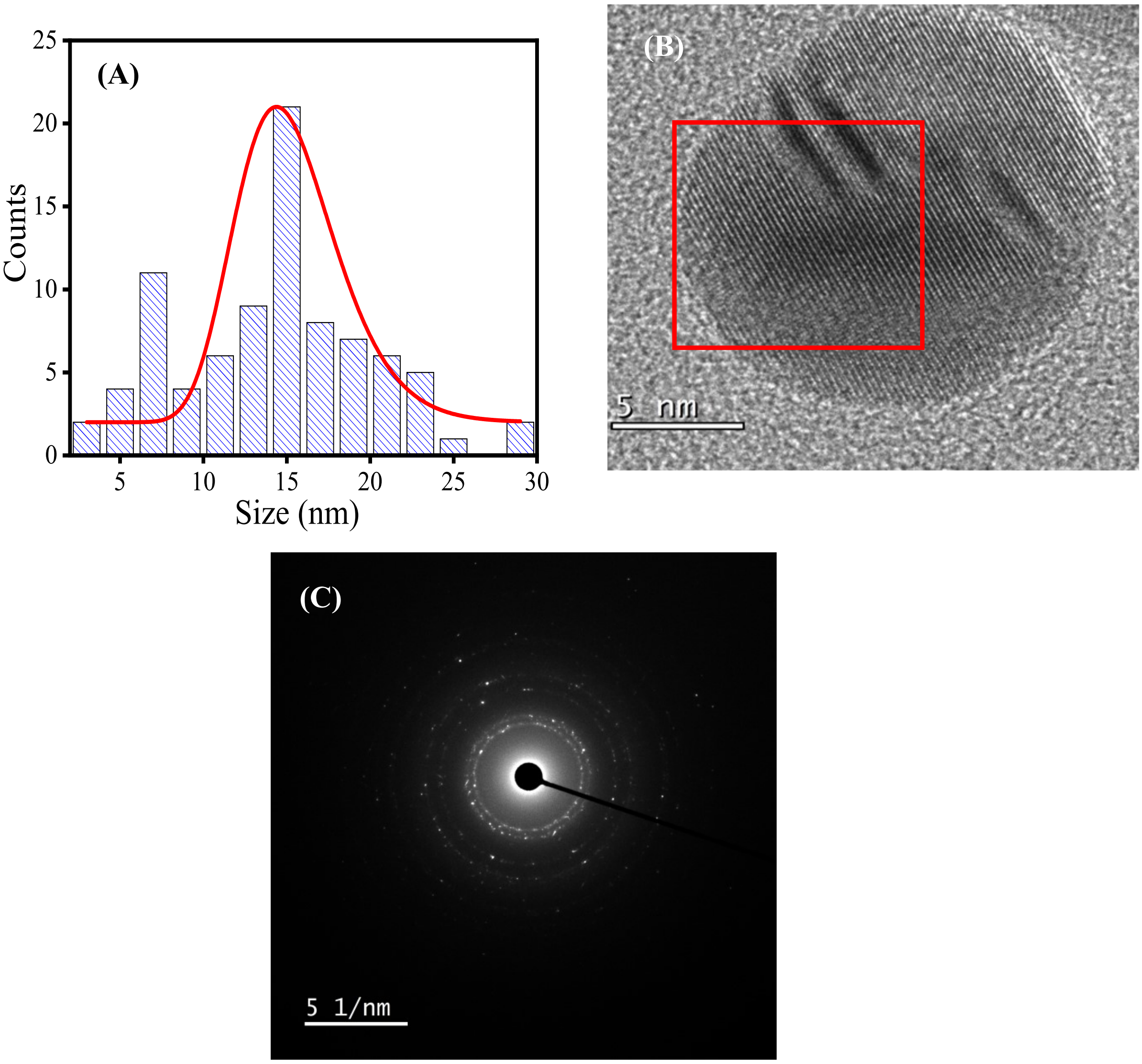

Fig. S1: (A) Particle size-distribution histogram; (B) HRTEM micrograph and (C) SAED pattern of HSP-AgNPs.

Determination of molar concentration of HSP-AgNPs

Assuming spherical shape of the nanoparticles and complete reduction of silver (I) to silver atoms, the molar concentration of HSP-AgNPs was calculated following the method described by Lewis *et al.* [22].

$$C = \frac{N_{NP}}{N_A} \quad (1)$$

Where C is the molar concentration of HSP-AgNPs, N_{NP} is the number of nanoparticles formed per 1 × 10^{-3} mol of $AgNO_3$, N_A is the Avogadro's number. The number of nanoparticles formed per 1 × 10^{-3} mol of $AgNO_3$ can be calculated using equation (*2*)

$$N_{NP} = \frac{N_{atom}}{N} \quad (2)$$

Where N_{atom} is the number of silver atoms and N is the number of silver atoms per nanoparticle.

The average number of silver atoms per nanoparticle (N) was calculated using *(3)*

$$N = \frac{\pi \rho D^3}{6\ M} \quad (3)$$

Where ρ is the density of silver (10.49 g/cm^3), M stands for atomic weight of silver (108 g/mol), D is the average diameter of silver nanoparticles (in nm).

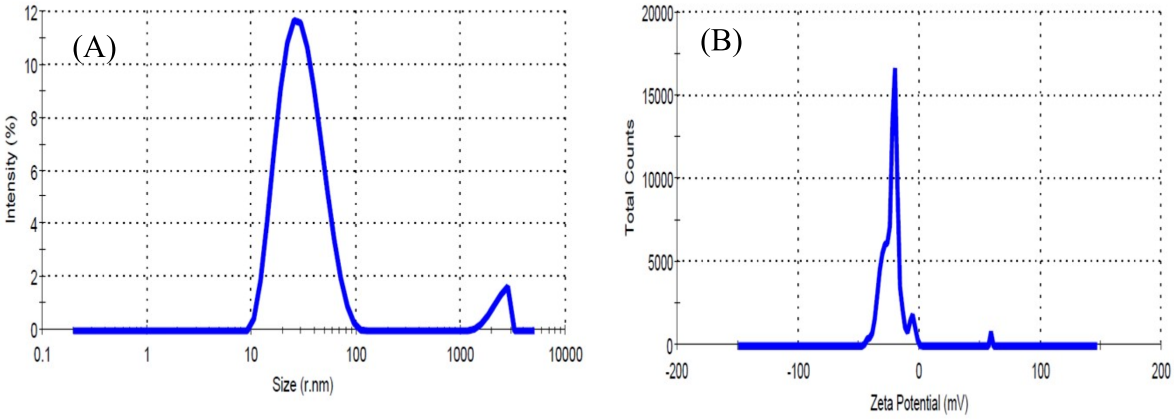

Fig. S2: (A) Particle size distribution (obtained from dynamic light scattering) and (B) Zeta potential of HSP-AgNPs.

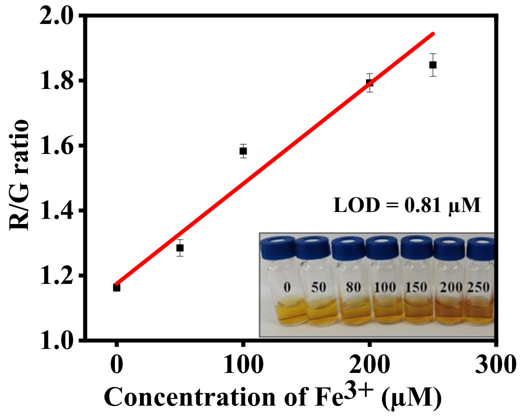

Fig. S3: Calibration curves for colorimetric sensing of $Fe^{3+}$by using HSP-AgNPs.

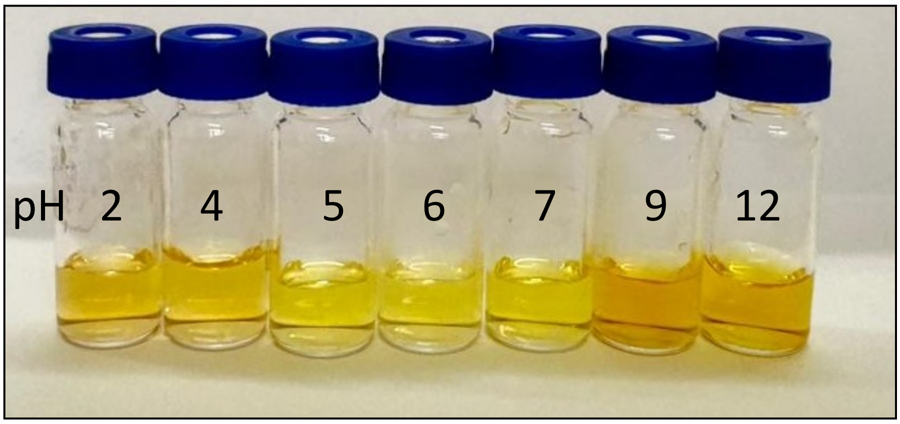

Fig. S4: The color of HSP-AgNPs at different pH.
